# *Streptococcus mutans* induces IgA nephropathy-like glomerulonephritis in rats with severe dental caries

**DOI:** 10.1038/s41598-021-85196-4

**Published:** 2021-03-11

**Authors:** Shuhei Naka, Kaoruko Wato, Taro Misaki, Seigo Ito, Daiki Matsuoka, Yasuyuki Nagasawa, Ryota Nomura, Michiyo Matsumoto-Nakano, Kazuhiko Nakano

**Affiliations:** 1 Department of Pediatric Dentistry, Okayama University Graduate School of Medicine, Dentistry and Pharmaceutical Sciences, Okayama, Japan; 2 Department of Pediatric Dentistry, Division of Oral Infection and Disease Control, Osaka University Graduate School of Dentistry, Suita, Osaka Japan; 3 Division of Nephrology, Seirei Hamamatsu General Hospital, Hamamatsu, Shizuoka Japan; 4 Department of Nursing, Faculty of Nursing, Seirei Christopher University, Hamamatsu, Shizuoka Japan; 5 Department of Nephrology and Endocrinology, National Defense Medical College, Tokorozawa, Saitama Japan; 6 Department of Internal Medicine, Division of Kidney and Dialysis, Hyogo College of Medicine, Nishinomiya, Hyogo Japan

**Keywords:** Microbiology, Bacteriology, Nephrology, Kidney diseases, Nephritis, Pathogenesis, Infection, Medical research, Experimental models of disease, Diseases, Dental diseases, Kidney diseases, Oral diseases

## Abstract

The mechanisms underlying immunoglobulin A nephropathy (IgAN), the most common chronic form of primary glomerulonephritis, remain poorly understood. *Streptococcus mutans*, a Gram-positive facultatively anaerobic oral bacterium, is a common cause of dental caries. In previous studies, *S. mutans* isolates that express Cnm protein on their cell surface were frequently detected in IgAN patients. In the present study, inoculation of Cnm-positive *S. mutans* in the oral cavities of 2-week-old specific-pathogen free Sprague–Dawley rats fed a high-sucrose diet for 32 weeks produced severe dental caries in all rats. Immunohistochemical analyses of the kidneys using IgA- and complement C3-specific antibodies revealed positive staining in the mesangial region. Scanning electron microscopy revealed a wide distribution of electron dense deposits in the mesangial region and periodic acid-Schiff staining demonstrated prominent proliferation of mesangial cells and mesangial matrix. These results suggest that IgAN-like glomerulonephritis was induced in rats with severe dental caries by Cnm-positive *S. mutans*.

## Introduction

*Streptococcus mutans* is a Gram-positive facultative anaerobic bacterium and a major causative agent of dental caries^1^. *S. mutans* occasionally induces infective endocarditis after invasion of the bloodstream during invasive dental procedures such as tooth extractions^2^. The *S. mutans* surface 120 kDa collagen-binding protein (Cnm) mediates adhesion to and invasion of vascular endothelial cells, which contributes to infective endocarditis^3,4^. The *cnm* gene is detected at a high frequency in *S. mutans*-positive heart valve specimens from patients with infective endocarditis^5^. Additionally, *cnm*-positive *S. mutans* strains are associated with deterioration of patients with systemic diseases such as cerebral haemorrhage, ulcerative colitis, and non-alcoholic steatohepatitis^6–11^.

Immunoglobulin A nephropathy (IgAN) is the most common chronic form of primary glomerulonephritis. Approximately 30%–40% of IgAN patients progress to end-stage kidney disease within 20 years^12–14^. The major clinical findings of IgAN are proteinuria and haematuria^15–17^, while common histopathological findings include proliferation of mesangial cells and mesangial matrix in the glomerulus^18^. Deposition of IgA and complement C3 in mesangial regions and electron dense deposits (EDDs) in the mesangial matrix are characteristic of IgAN^19^. However, the detailed pathogenesis of the disease is poorly understood.

IgAN patients often show deterioration of macroscopic haematuria in mucosal infections^19^. Immune dysregulation in the upper airway mucosa due to infection by *Escherichia coli*, *Pseudomonas aeruginosa, Haemophilus parainfluenzae*, and methicillin-resistant *Staphylococcus aureus* is believed to cause glomerular tissue damage^20–22^. Several case reports have described prior infection of the upper airway mucosa in patients with acute IgAN^23^. A recent review has also suggested that mucosal alterations such as infections activate the innate immune system, aggravate pre-existing IgAN, and promote disease manifestations such as macrohaematuria^21^.

Periodontal bacteria such as *Treponema* spp. and *Campylobacter rectus* have been implicated in the development of IgAN^24,25^ and a microbiome study has shown that changes in the subgingival microbial structure and IgAN correlate in patients with chronic periodontitis^26^, although the underlying mechanisms remain unclear. In some studies, nephritis has been induced in rabbits by intravenous administration of *S. mutans* proteins^27^. Recently, we found Cnm-positive *S. mutans* at a high frequency in the oral cavities of IgAN patients^28^. Additionally, IgAN patients that harbour Cnm-positive *S. mutans* show significantly higher numbers of caries-experienced teeth and more severe proteinuria^29^.

Our recent study demonstrated that intravenous administration of Cnm-positive *S. mutans* transiently induces an IgAN-like condition in rats^30^. In the present study, we assessed whether IgAN-like conditions were also observed in a rat model of dental caries.

## Results

### Development of a rat model of severe dental caries

We used a streptomycin-resistant Cnm-positive strain isolated from an IgAN patient (SN74R) and a streptomycin-resistant Cnm-negative strain isolated from a healthy child (MT8148R) to evaluate the intensity of dental caries in rats. Our first attempts using a conventional strategy of feeding for 11 weeks produced only mild dental caries. Thus, we fed the rats for extended periods of 16, 24, 32, and 40 weeks in an attempt to model severe dental caries. Rats fed for 16 weeks showed no lesions that extended to the pulp space, whereas 60% of rats fed for 24 weeks showed severe dental caries that extended to the pulp space. All rats fed for 32 weeks showed severe dental caries that extended to the pulp space. Rats fed for 40 weeks showed more severe dental caries lesions, although the status of dental caries could not be evaluated because the tooth crown was completely destroyed. Therefore, we used a maximum feeding period of 32 weeks. Rats were fed for 16, 24, and 32 weeks and then kidney tissues were collected, which showed that the rates of IgA nephropathy-like glomerulonephritis lesions evaluated by histopathological and immunochemical staining using an anti-IgA antibody were 4.2% (1/24), 26.1% (6/23,) and 51.7% (15/29), respectively. Thus, we used a feeding period of 32 weeks for the following experiments.

### Oral conditions after treatment

**Figure 1:**
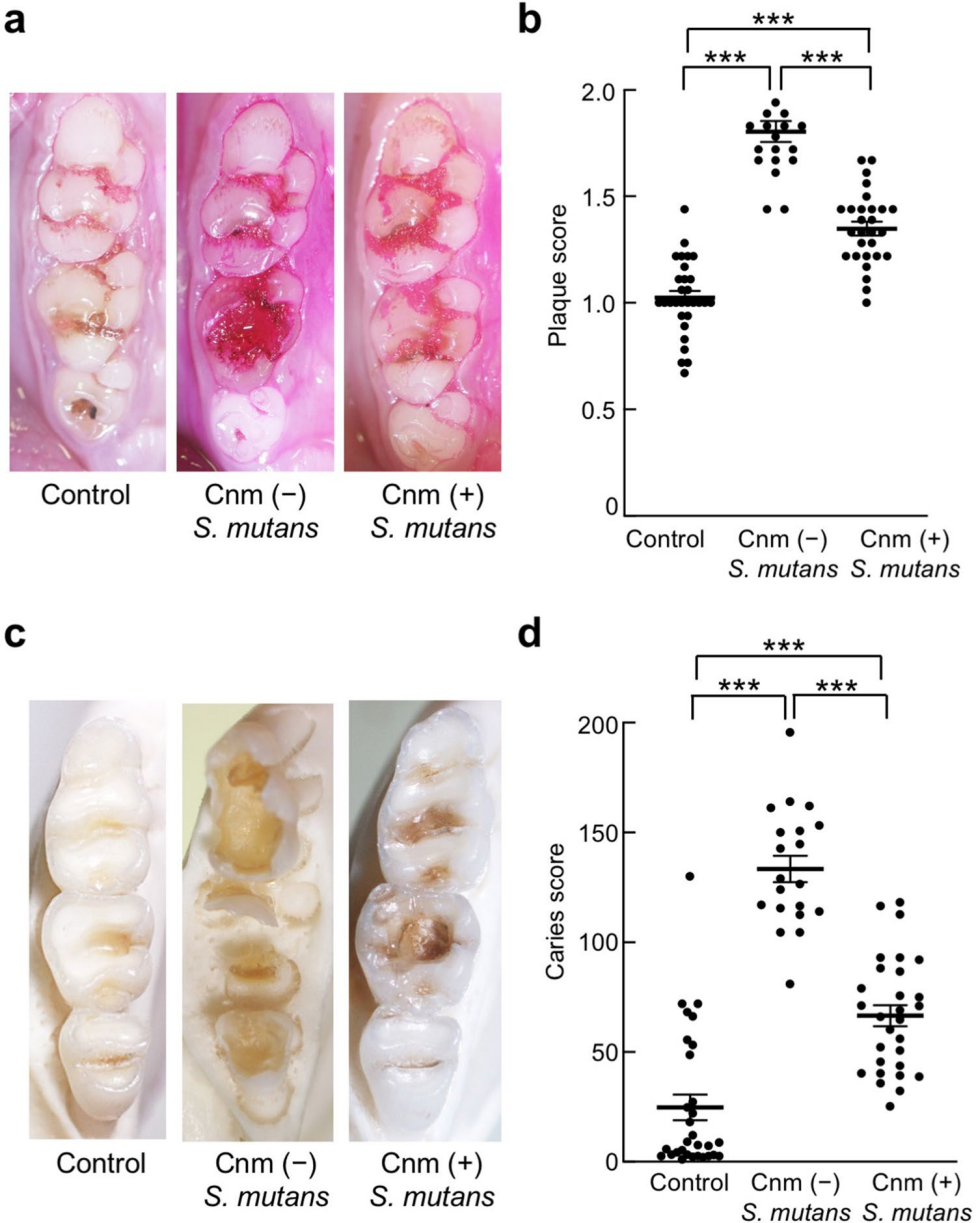
Plaque and caries scores. Each column represents the mean ± standard error of the mean of the control group (n = 30), Cnm (−) *S. mutans* group (n = 20), or Cnm (+) *S. mutans* group (n = 29). Statistical significance was determined using he Kruskal–Wallis test. ***P < 0.001.

In rats fed for 32 weeks, dental plaque accumulation was prominent in both the Cnm-positive group (SN74R) and Cnm-negative group (MT8148R) compared with the control group (Fig. 1a). The dental plaque scores of rats inoculated with Cnm-negative *S. mutans* were the highest, followed by rats inoculated with Cnm-positive *S. mutans*. The scores of both groups were significantly greater than those of the control group (*P* < 0.001) (Fig. 1b). Only attrition and slight dental caries were observed in the control group, whereas severe dental caries were observed in Cnm-positive and Cnm-negative groups (Fig. 1c). The dental caries scores of the Cnm-positive and Cnm-negative groups were both significantly greater than those of the control group (*P* < 0.001) (Fig. 1d).

### Systemic conditions after treatment

Urinary protein values in rats inoculated with Cnm-positive *S. mutans* were not significantly different from those of rats inoculated with Cnm-negative *S. mutans* or control rats. Conversely, haematuria was more frequent in the Cnm-positive group compared with Cnm-negative and control groups (*P* < 0.001) (Table 1). There were no significant differences in the serum levels of albumin (ALB), blood urea nitrogen (BUN), or creatinine (CRE) among the groups (Supplementary Table 1).

**Table 1:** Analysis of urine components in rats inoculated with Cnm-positive and Cnm-negative *S. mutans*.

Groups	Clinical parameters	
	Urinary protein ratio (mean ± SEM)	Haematuria (N, %)
Control (N = 30)	1.02 ± 0.13^a^	0/30 (0)^d^
Cnm (−) *S. mutans* (N = 20)	0.67 ± 0.10^b^	0/20 (0)^e^
Cnm (+) *S. mutans* (N = 29)	0.97 ± 0.20^c^	9/29 (31.0)^f^

^a–^^c^Statistical significance was determined using analysis of variance with Bonferroni’s correction. ^d–f^Statistical significance was determined using Fisher’s exact test (^d,f^*P* < 0.001 and ^e,f^*P* < 0.001). SEM, standard error of the mean.

### Immunohistochemical analyses of kidney tissues

**Figure 2:**
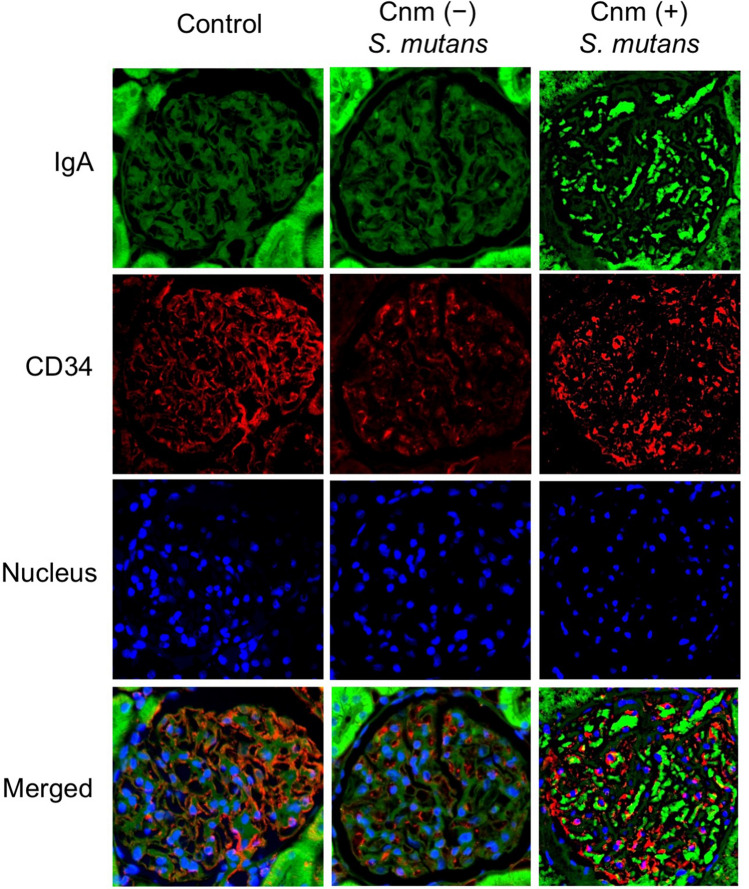
Representative histopathological appearance of kidney tissues by immunohistochemistry with an IgA-specific antibody. The first image shows staining with an anti-IgA antibody. The second image shows staining with an anti-CD34 antibody. The third image shows nuclear staining. The fourth row of images is superpositions of the top three images.

**Figure 3:**
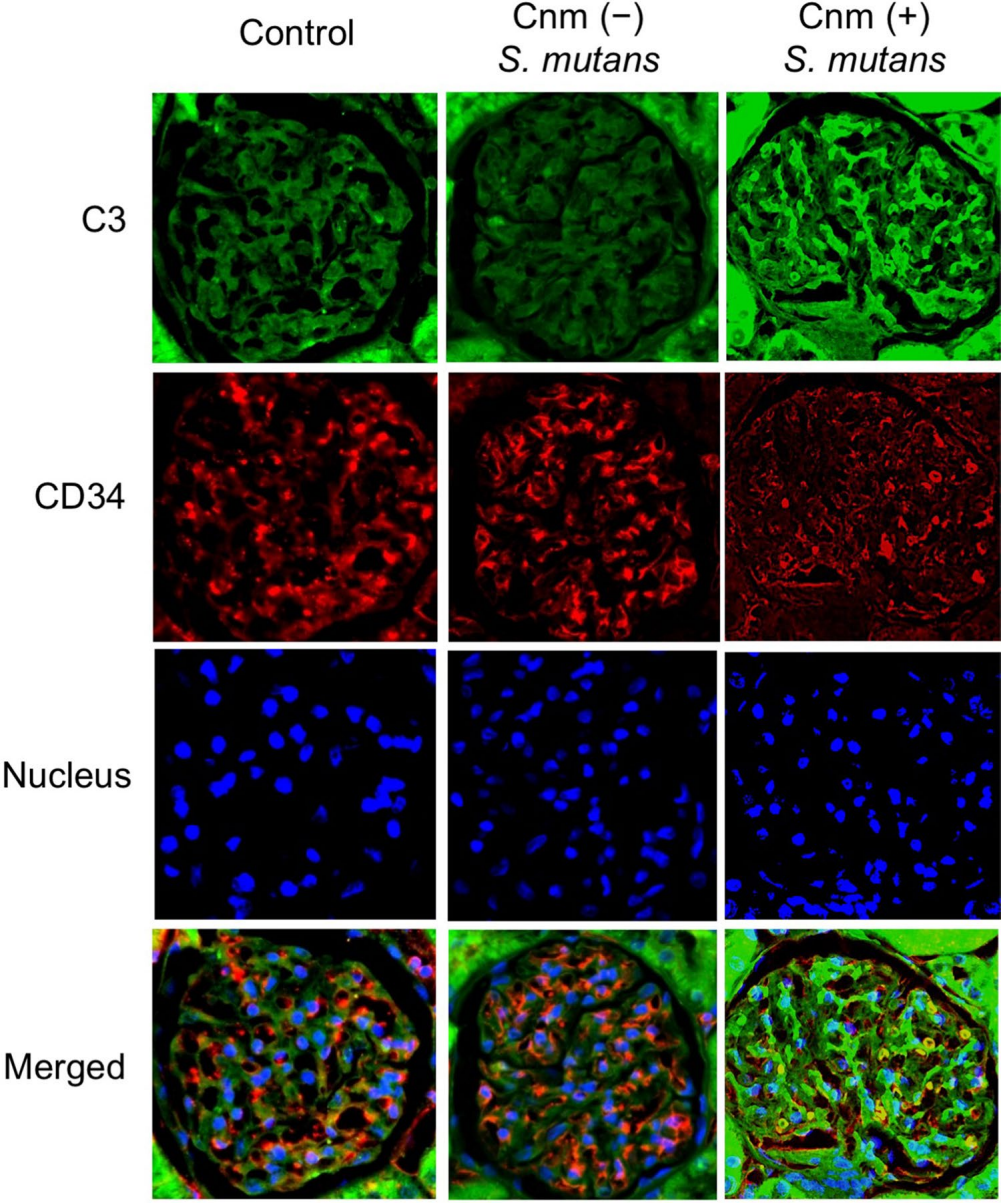
Representative histopathological appearance of kidney tissues by immunohistochemistry with a C3-specific antibody. The first image shows staining with an anti-C3 antibody. The second image shows staining with an anti-CD34 antibody. The third image shows nuclear staining. The fourth row of images is superpositions of the top three images.

**Figure 4:**
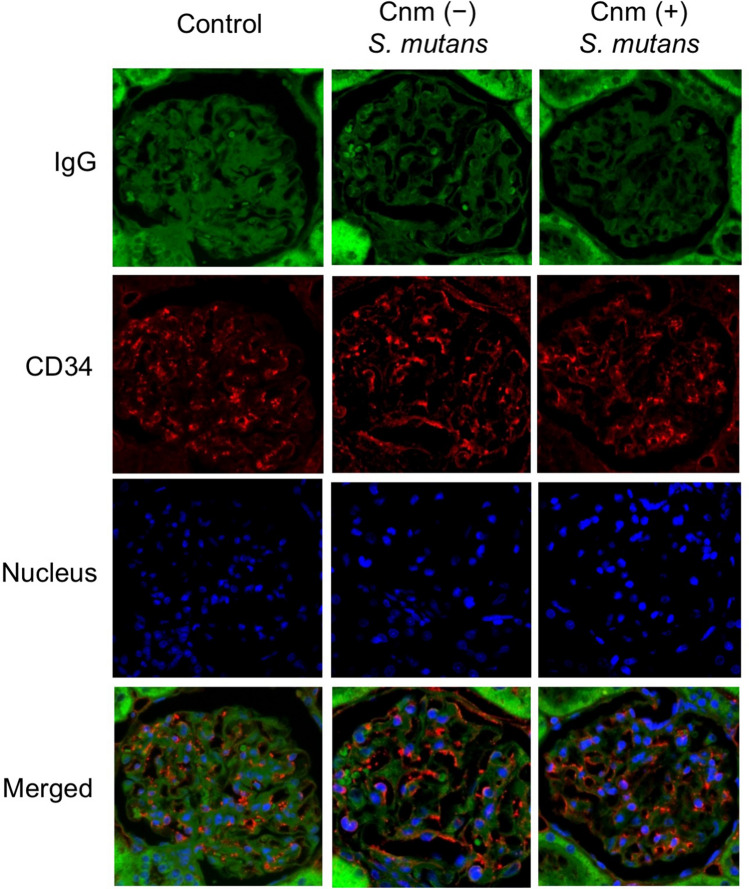
Representative histopathological appearance of kidney tissues by immunohistochemistry with an IgG-specific antibody. The first image shows staining with an anti-IgG antibody. The second image shows staining with an anti-CD34 antibody. The third image shows nuclear staining. The fourth row of images is superpositions of the top three images.

**Figure 5:**
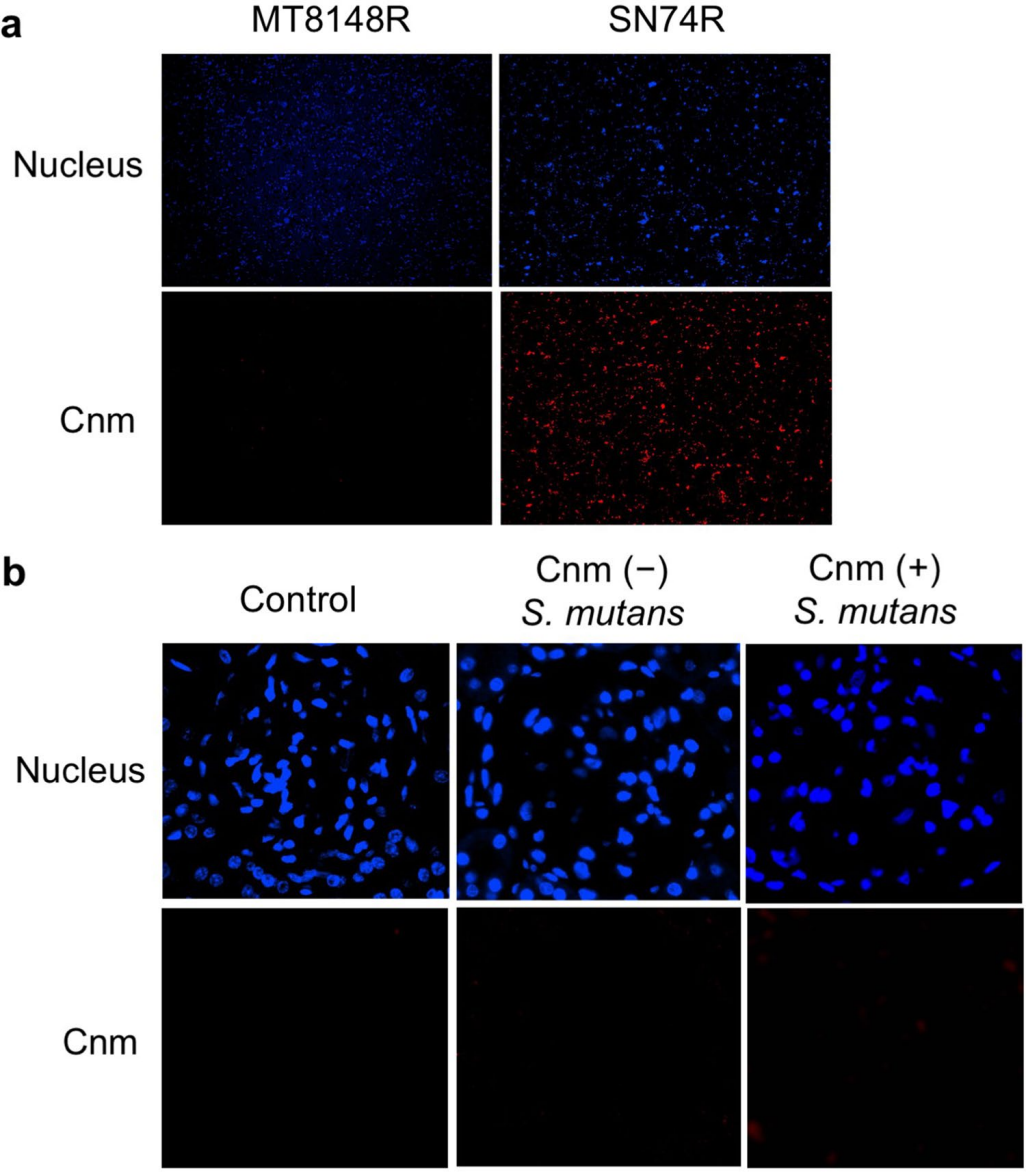
Representative histopathological appearance of bacteria and kidney tissues by immunohistochemistry with a Cnm-specific antibody. The first image shows nuclear staining. The second image shows staining with an anti-Cnm antibody. (**a**) Bacteria. (**b**) Kidney tissues.

Immunohistochemical analyses using IgA- and C3-specific antibodies showed prominent positive reactions in the mesangial regions of rats inoculated with Cnm-positive *S. mutans* (Figs. 2, 3). The positive staining rates for IgA, C3, and their combination in the Cnm-positive group were significantly higher than those in Cnm-negative and control groups (Table 2). Immunochemical analyses using an IgG-specific antibody revealed no IgG deposition in any of the rats (Fig. 4). Additionally, immunochemical analyses using a Cnm-specific antibody revealed no Cnm deposition in the kidneys of any rats, although Cnm was stained in Cnm-positive *S. mutans* SN74R, but not in Cnm-negative *S. mutans* MT8148 (Fig. 5).

**Table 2:** Frequencies of positive immunohistochemical staining in rats inoculated with Cnm-positive and Cnm-negative *S. mutans*.

Groups	Targets		
	IgA (%)	C3 (%)	IgA and C3 (%)
Control (N = 30)	1/30 (3.3)^a^	3/30 (10.0)^d^	1/30 (3.3)^g^
Cnm (−) *S. mutans* (N = 20)	1/20 (5.0)^b^	1/20 (5.0)^e^	1/20 (5.0)^h^
Cnm (+) *S. mutans* (N = 29)	15/29 (51.7)^c^	12/29 (41.4)^f^	12/29 (41.4)^i^

Statistical significance was determined using Fisher's exact test. (^a,c^*P* < 0.001, ^b,c^*P* < 0.001, ^d,f^*P* < 0.01, ^e,f^*P* < 0.01, ^g,i^*P* < 0.001, and ^h,i^*P* < 0.01).

### Histopathological analyses of kidney tissues

**Figure 6:**
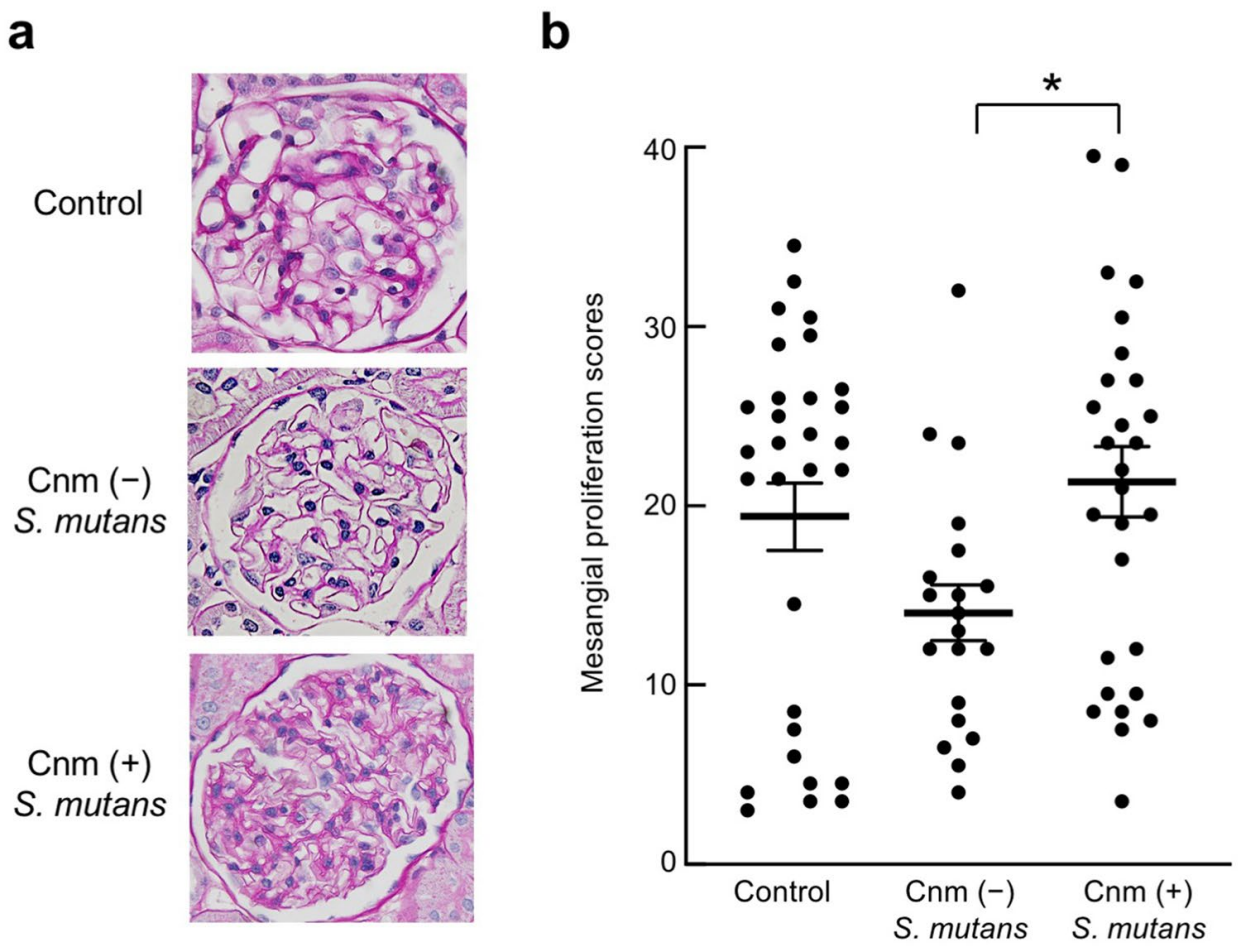
Histopathological appearance of kidney tissues after PAS staining. Mesangial proliferation scores of rats inoculated with Cnm-positive and Cnm-negative *S. mutans* were compared. Each column represents the mean ± standard error of the mean of the control group (n = 30), Cnm (−) *S. mutans* group (n = 20), or Cnm (+) *S. mutans* group (n = 29). Statistical significance was determined using analysis of variance with Bonferroni’s correction. *P < 0.05.

**Figure 7:**
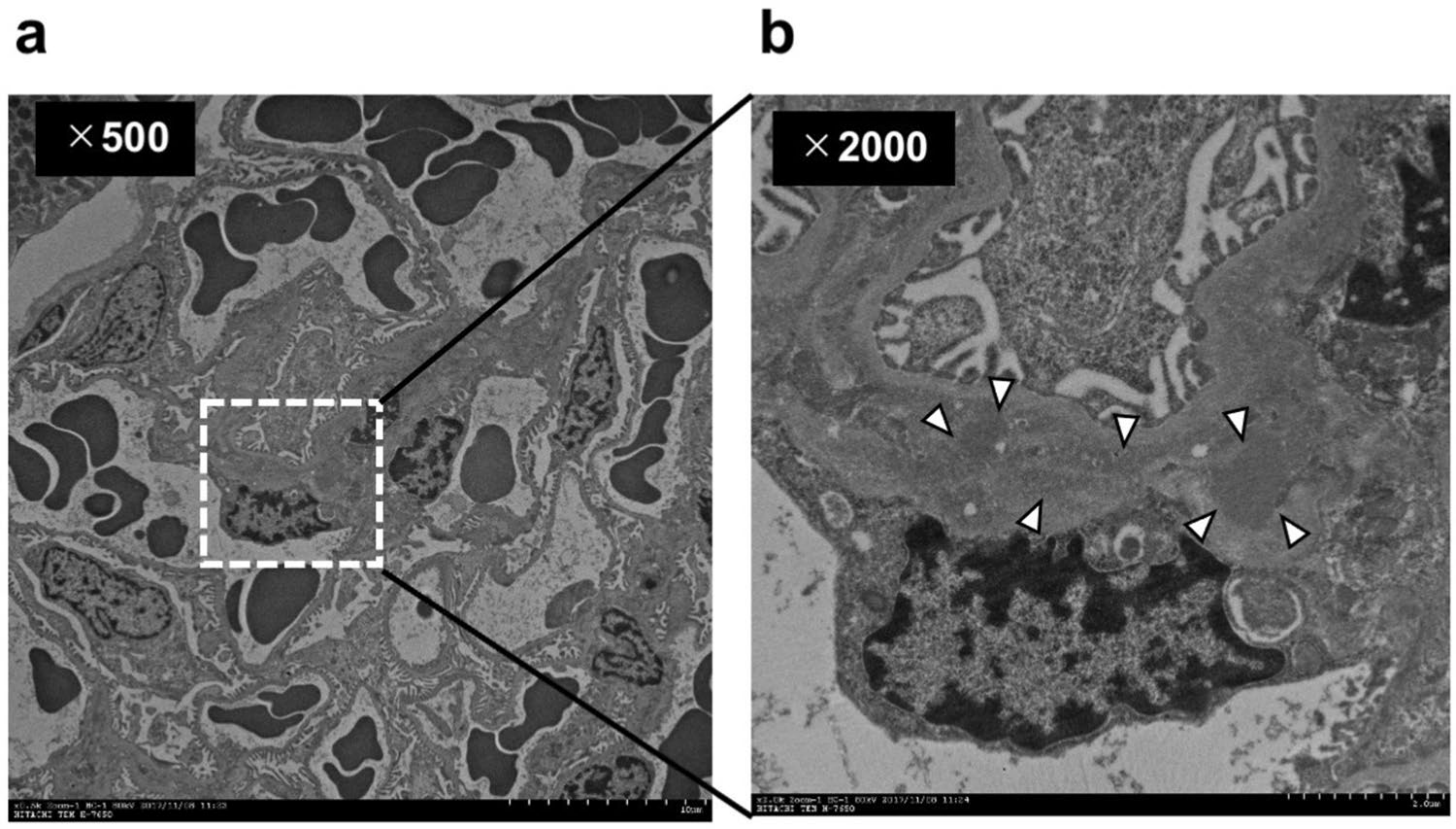
Representative appearance of kidney tissue by transmission electron microscopy in rats inoculated with *S. mutans*. (**a**, **b**) Representative appearance of the kidneys of rats inoculated with *S. mutans*. White arrows indicate electron dense deposits (EDDs).

Histopathological analyses of period acid-Schiff (PAS)-stained sections revealed prominent proliferation of mesangial cells and mesangial matrix in rats inoculated with Cnm-positive *S. mutans* (Fig. 6a). The mesangial proliferation scores of the Cnm-positive group were significantly greater than those of the Cnm-negative group (*P* < 0.05) (Fig. 6b). Scanning electron microscopy revealed a wide distribution of EDDs in mesangial regions (Fig. 7).

### Comparison of IgA-positive and IgA-negative samples from rats inoculated with Cnm-positive *S. mutans*

**Figure 8:**
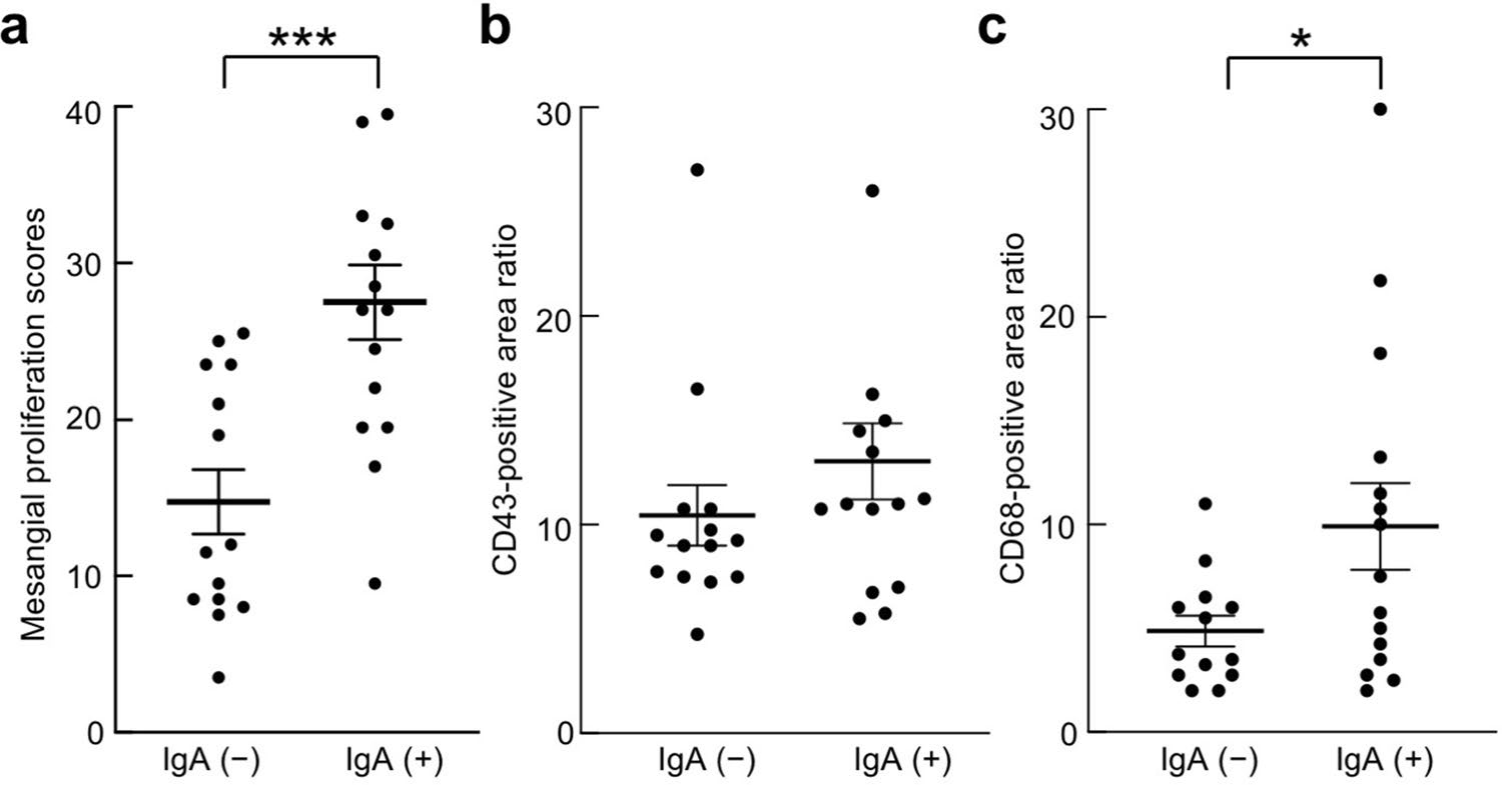
Comparison of IgA-positive and IgA-negative samples from rats inoculated with Cnm-positive *S. mutans*. (**a**) Mesangial proliferation scores, (**b**) CD43-positive areas, and (**c**) CD68-positive areas were compared in IgA-positive and -negative samples from the Cnm (+) *S. mutans* group. Statistical significance was determined using the Student t-test. *P < 0.05, ***P < 0.001.

The mesangial proliferation scores of IgA-positive samples were significantly greater than those of IgA-negative samples (*P* < 0.001) (Fig. 8a). There was no significant difference in the sizes of CD43-positive areas between the two groups (Fig. 8b). The CD68-positive areas of IgA-positive samples were significantly greater than those of IgA-negative samples (*P* < 0.05) (Fig. 8c).

## Discussion

Recently, we demonstrated that intravenous administration of Cnm-positive *S. mutans* to rats causes transient IgAN-like symptoms^30^. This model simulates the entry of large numbers of bacteria into the bloodstream during invasive dental procedures such as tooth extractions. Interestingly, we found IgAN-like lesions in the rat model of severe dental caries in the present study. To the best of our knowledge, this is the first study to demonstrate IgAN-like lesions in a rat model of dental caries.

In the conventional rat model of dental caries, rats are fed for 11 weeks and inoculated with a standard strain of *S. mutans* (MT8148R), which results in mean dental caries scores of approximately 50^31–36^. In our study, the mean caries score of rats inoculated with MT8148R (Cnm negative) and fed for 32 weeks was approximately 130. In rats inoculated with SN74R (Cnm positive), the average caries score was approximately 80. These results showed that MT8148R (Cnm negative) causes more severe dental caries lesions than SN74R (Cnm positive), whereas SN74R (Cnm positive) causes dental caries with a greater intensity.

Clinical findings in IgAN patients include persistent microscopic haematuria as a mandatory finding and intermittent or persistent urinary protein as a frequent finding^37,38^. The presence of microscopic haematuria is necessary for diagnosis, especially in the early stages of disease or during active periods of IgAN^39,40^. In fact, haematuria was identified in rats fed for 32 weeks and inoculated with Cnm-positive *S. mutans* at rates significantly higher than those of Cnm-negative and control groups. However, urinary protein was normal in all groups. IgAN can decrease renal functions at approximately 10 years after becoming chronic^14^. Kidney dysfunction is associated with the development of nephritis with a subsequent decrease in serum ALB and increases in BUN and CRE^41^. In our rat model of dental caries, there were no significant differences in the levels of any serum parameter or proteinuria among the groups. These results suggest that nephritis, which includes significant renal dysfunction, did not occur after 32 weeks of feeding and *S. mutans* inoculation. Because the mesangial proliferation scores also showed that nephritis was very weak, we concluded that rats fed for 32 weeks reached the early stages of IgAN.

More than 90% of IgAN patients show deposition of complement C3 in glomeruli^42^. Most C3 is found in mesangial and paramesangial regions including some vascular endothelial cells^19,43^. Additionally, patients with IgG coprecipitation show faster progression of renal dysfunction, which is a potential risk factor for a poor prognosis^44^. In the present study, both IgA and complement C3 were deposited in the Cnm-positive group and their positive rates were significantly higher than those of the other groups. IgA and complement C3 were both mainly deposited in the mesangial region. These results suggested that the rat caries model in this study simulated the histopathological features of IgAN, while no IgG deposition was observed in any group.

Typical histopathological findings in IgAN include the presence of increased numbers of mesangial cells and matrix in the mesangial region as well as deposition of immune complexes^45^. Mesangial cell proliferation is defined as the presence of more than four cells in a single mesangial region^18^. In the present study, the numbers of mesangial cells and the extent of matrix proliferation in the glomerulus were observed and evaluated using optical microscopy. The mesangial proliferation scores of the Cnm-positive group were significantly higher than those of the Cnm-negative group. In the Cnm-positive group, there was clear proliferation of mesangial cells and increased matrix in the mesangial region. Additionally, IgA and complement C3 deposition was observed in the glomerular mesangial region by immunostaining and EDDs were observed in the mesangial region in the Cnm-positive group. These histopathological findings suggested that the typical characteristics of IgAN were present in the mesangial region.

It has been reported that macrophages mobilised in the glomerulus are involved in the development of IgAN and exacerbation of the disease state^46–48^. In paediatric cases of IgAN, macrophage infiltration into the glomerulus was clinically correlated with the extent of haematuria^49^. In the present study, we examined several factors associated with the progression or exacerbation of IgAN in specimens from the Cnm-positive group that showed IgA deposition. We assessed mesangial proliferation scores, CD43 positivity (a marker of neutrophil infiltration), and CD68 positivity (a marker of macrophage infiltration). Mesangial proliferation scores and CD68-positive rates in the IgA-positive group were significantly higher than those in the IgA-negative group. However, there was no significant difference in the CD43-positive rate between the groups. These results revealed that, among rats inoculated with Cnm-positive *S. mutans*, IgA deposition in the glomerulus was associated with abnormalities such as increased mesangial cells and mesangial matrix as well as increased macrophage infiltration. These findings suggested that the pathogenesis of our rat model of dental caries was similar to that of IgAN in terms of histopathology.

The present study demonstrated that specific *S. mutans* in the oral cavity induced IgA nephropathy-like glomerulonephritis in rats. Although the phenomenon was shown clearly, specific factors correlated to the disease remain to be elucidated. Our previous studies have indicated that Cnm may be an important factor^28,29^. In fact, rats with Cnm-positive strain SN74 in their oral cavity had the disease, whereas those with Cnm-negative strain MT8148 did not in the present study. However, not only Cnm, but also other cell surface proteins are different because these two strains are natural isolates. Therefore, it is reasonable to speculate that Cnm may be the most important factor, but it is also possible that other unknown factors may be related to the development of the disease. Therefore, further studies should focus on this point.

It is important to clarify the mechanism of IgAN-like glomerulonephritis caused by *S. mutans* in the present study. We have previously reported that Cnm-positive *S. mutans* strains from the oral cavity are associated with urinary protein levels in IgAN patients, especially those with a high dental caries status^29^. We have also reported that Cnm-positive *S. mutans* strains in the tonsils are associated with severe IgAN and the Cnm protein itself does not have direct effects on the kidney^50^. In the present study, immunostaining using the anti-Cnm antibody showed that none of the kidney specimens were positive, which indicated that the Cnm protein itself did not have direct effects on the kidney. Therefore, it is reasonable to speculate that there was no direct reaction to the bacterium itself, but immune disorders caused by *S. mutans* in the oral cavity or spleen and other organs, which the bacteria reach via the bloodstream, might be a possible cause.

A recent microbiome study has shown that changes in the subgingival microbial structure and IgAN correlate in patients with chronic periodontitis^26^. Furthermore, a recent review has suggested that mucosal alterations such as infections activate the innate immune system, aggravate pre-existing IgAN, and promote disease manifestations such as macrohaematuria^21^. Taken together, frequent and repeated Cnm-positive *S. mutans* immunoreactions with IgA in mucosal tissues, such as tonsils in the oral cavity or the spleen and others, might induce a glycosylation defect in serum IgA1 molecules, which plays an important role in the pathogenesis of IgAN. This is because glycosylation deficiency in IgA1 molecules is a primary pathogenesis of IgAN^19,51^. Further studies are required to elucidate the detailed mechanisms.

In summary, we hypothesised that Cnm-positive *S. mutans* harboured in the oral cavity induced immunoreactions that potentially led to the occurrence of IgAN-like lesions in rats. Compared with the histopathological and immunohistochemical findings in a rat model of intravenous *S. mutans* administration^30^, IgAN-like findings were more prominent in the rat model of severe dental caries used in the present study. We expect that this novel potential mechanism might contribute to solving the many problems associated with this intractable disease.

## Methods

### *S. mutans* strains and culture medium

*Streptococcus mutans* strain SN74 (serotype *e*) was isolated from the oral cavity of a patient with severe IgAN^30^. The strain isolation was conducted in full adherence to the Declaration of Helsinki. Study protocols were approved by the Ethics Committee of Seirei Hamamatsu General Hospital (1486) and Osaka University Graduate School of Dentistry (H25-E24)^28^. The subject was informed of the protocols and gave their written consent prior to participation. The SN74 strain was positive for cell surface Cnm. The Cnm-negative *S. mutans* strain MT8148 (serotype *c*) is an oral isolate from a Japanese child and is widely used as a reference strain^31^. These strains were cultured on Mitis-Salivarius agar (Difco Laboratories, Detroit, MI, USA) plates containing bacitracin (0.2 U/mL; Sigma Chemical Co., St. Louis, MO, USA) or in brain heart infusion (BHI; Difco) broth.

### Rat model of dental caries

All rats were treated humanely in accordance with the National Institutes of Health and AERI-BBRI Animal Care and Use Committee guidelines. Experiments using the rat caries model were approved by the Committee on Animal Experiments of the Graduate School of Dentistry, Osaka University (approval number: Animal-25-020-0) and the Committee on Animal Experiments of Okayama University (approval number: OKU-2017213). The rat model of severe dental caries followed the method of Matsumoto-Nakano et al.^36^ with some modifications as follows. Streptomycin-resistant (1500 μg/mL) derivatives of strains SN74 (Cnm positive) and MT8148 (Cnm negative) were generated by sequential transfer in Mitis-Salivarius agar containing 500, 1000, and 1500 µg/mL streptomycin and named SN74R and MT8148R, respectively. Both strains were adjusted to an OD550 of 1.0 [equivalent to 1 × 10^9^ colony-forming units (CFU)/mL] with phosphate-buffered saline (PBS) after incubation for 18 h in 10 mL BHI medium at 37 °C. The cells were concentrated 10 and 100 times, which yielded solutions equivalent to 1 × 10^10^ and 1 × 10^11^ CFU/mL, respectively.

Specific pathogen-free Sprague–Dawley rats (male, 2 weeks old; CLEA Japan, Tokyo, Japan) were provided with sterile distilled water containing penicillin (4000 U/mL) (Meiji Seika Pharma Co, Tokyo, Japan) and fed freely. A regular diet (Rodent Diet CE-2, CLEA Japan) was mixed with tetracycline hydrochloride (4 mg/mL) (FUJIFILM Wako Pure Chemical Co, Osaka, Japan) and provided for 2 days. The rats were then divided into three groups: control (n = 30), SN74R-inoculated (n = 29), and MT8148R-inoculated (n = 20) groups. SN74R and MT8148R strains were administered directly into the oral cavity using a pipette once a day for 5 days in SN74R and MT8148R groups, respectively. However, no administration was performed in the control group. All rats were fed a caries-inducing diet containing 56% sucrose (Diet 2000, CLEA Japan) and maintained until 34 weeks of age.

### Evaluation of dental plaque and caries

Excised maxillary bones were rinsed with dental plaque staining solution (Sunstar Inc., Osaka, Japan). The jawbones were then observed under a stereomicroscope using the method of Regolati and Hotz^52^. Dental plaque adherence to buccal, occlusal and palatal surfaces was scored from 0 to 4 (Score 0: no staining; Score 1: stained 1/4 of the area or less; Score 2: stained 1/2 of the area or less, but more than 1/4 of the area; Score 3: stained 3/4 of the area or less, but more than 1/2 of the area; Score 4: stained more than 3/4 of the area) and the mean in each group was calculated. Additionally, the maxilla and mandibles were subjected to heat treatment in an autoclave (TOMY SEIKO Co., Ltd, Tokyo, Japan) at 121 °C under two atmospheres of pressure for 1 and 3 min, respectively, and then soft tissues were removed. Jawbone caries were assessed in accordance with the description of Keyes^53^, which was modified for molars in rats on the basis of the method of severe dental caries in hamsters, under a stereomicroscope. The method divides into 94 similar areas on the surface of all molar teeth and evaluates the intensity of destruction as 0 (none), 1.5 (half), and 3 (entire). The dental caries scores are expressed as the sum of the product of the area and intensity. The maximum score for complete destruction of all molar teeth is 282.

### Analyses of urinary and serum components

Urine and serum were analysed in accordance with the method of Naka et al.^30^. At 34 weeks of age, the rats were transferred to a sterile plastic container and urine was collected by pipetting into a sterile tube. The precipitate and supernatant were separated by centrifugation at 4 °C for 3 min at 180×*g*. To assess the presence or absence of haematuria, 10 μL of sterile distilled water was added to the sediment and mixed by pipetting. Smears on glass slides were fixed with 100% methanol (FUJIFILM Wako Pure Chemical Co.). After drying, the smears were stained with a Giemsa solution (FUJIFILM Wako Pure Chemical Co.) for 10 min. After staining, the smears were washed with water, dried, and observed under an optical microscope (BX53F, Olympus Co., Tokyo, Japan) at ×400 magnification. To assess haematuria, the presence of 10 or more red blood cells in one visual field was considered positive^54^. After anaesthesia induced by inhalation of isoflurane (Pfizer Japan Inc, Tokyo, Japan)^55^, 0.75 mL medetomidine hydrochloride (ZENOAQ, Fukushima, Japan), 2 mL midazolam (Astellas Pharma, Tokyo, Japan), and 2.5 mL butorphanol tartrate (Meiji Seika Pharma Co) were added to 45 mL sterile distilled water. General anaesthesia was induced in rats by intraperitoneal administration at a dose of 0.1 mL per 10 g body weight. Then, blood was collected and serum was prepared by centrifugation at 1600×*g* for 10 min at 4 °C. Urinary levels of protein and CRE were measured by Nagahama Lifescience (Oriental Yeast Co., Ltd, Shiga, Japan). Serum levels of CRE, ALB, and BUN were also measured by Nagahama Lifescience.

### Histological evaluation of kidneys

Histological evaluation of kidneys was performed by following the method of Ito et al.^50^ and Naka et al.^30^. Excised kidney tissue was fixed with 3.7% formaldehyde (FUJIFILM Wako Pure Chemical Co.) in PBS and then embedded in paraffin to prepare 3-μm-thick tissue sections. The sections were subjected to PAS staining in accordance with the method of Schaart et al.^56^ and observed under an optical microscope (BX53F, Olympus) to assess the proliferation of glomerular mesangial cells and mesangial matrix in glomeruli. Mesangial proliferation scores were calculated on the basis of the percentage of 50 glomeruli with mesangial cell and matrix proliferation in PAS-stained sections^30^. Alterations of IgA, C3, IgG, CD34, and Cnm expression in tissues were detected using standard immunohistochemical techniques witCD34 (vascular endothelial cell marker)-, and Cnm-specific antibodies. The primary antibodies were mouse anti-rat IgA (BD Biosciences, Franklin Lakes, NJ, USA), anti-C3 (B-9) (sc-28294; Santa Cruz Biotechnology, Dallas, TX, USA), anti-rat IgG2a (BioLegend, CA, USA), and anti-CD34 (EP373Y) (ab81289; Abcam, Cambridge, UK). The secondary antibodies were donkey anti-mouse IgG H&L (Alexa Fluor 488) preadsorbed (ab150109; Abcam) and donkey anti-rabbit IgG H&L (Alexa Fluor 647) (ab150075; Abcam). Indirect immunofluorescence was performed using these antibodies. Stained sections were observed under an all-in-one fluorescence microscope (BZ-X700; Keyence, Osaka, Japan). The distribution of immune cells in the kidney was analysed by assessing CD43 (neutrophil marker) and CD68 (macrophage marker) expression in the tissues using standard immunohistochemical techniques with CD43- and CD68-specific antibodies. The primary antibodies were mouse anti-rat CD43 (W3/13) (BD Biosciences) and anti-CD68 (ED1) (BD Biosciences). Histofine simple stain rat MAX-PO (M; Nichirei Biosciences Inc., Tokyo, Japan) was used as the secondary antibody. Twenty glomeruli were selected in random images obtained at low magnification and the percentages of glomeruli with positive staining for each antibody were determined. Values for *S. mutans* groups were calculated as the ratio to the average scores of the control group, which were equivalent to 1.0 at each time point. Images were analysed using ImageJ software (National Institutes of Health) to determine the ratios of CD43- and CD68-positive areas to the whole glomerulus.

### Transmission electron microscopy

Transmission electron microscopy was performed in accordance with the method of Naka et al.^30^. For pre-fixation, excised kidney tissue specimens were immersed in 0.1 M PBS (pH 7.4) containing 2% glutaraldehyde and 2% paraformaldehyde for 16–18 h. Post-fixation was performed in 2% osmium tetroxide for 1.5 h. After washing with PBS, the specimens were dehydrated in a graded ethanol series and embedded in low viscosity resin (Spurr resin; Polysciences). Then, 80-nm ultrathin sections were prepared using an ultramicrotome (EM-UC 7; Leica, Tokyo, Japan) and stained with uranyl acetate and lead citrate. The specimens were then observed under a transmission electron microscope (H-7650; Hitachi, Tokyo, Japan).

### Statistical analyses

Statistical analyses were performed using GraphPad Prism 8 (Graph Pad Software Inc., San Diego, CA, USA). All data are presented as means ± standard errors of the means. Differences in dental plaque and caries scores were assessed using the Kruskal–Wallis test. Differences in whole body weights, serum parameters, mesangial proliferation scores, and CD43- and CD68-positive areas were assessed using analysis of variance with Bonferroni’s correction. Differences in urinary protein were assessed by Thompson’s rejection test, followed by Fisher’s exact probability test. Positive immunohistochemical staining was compared using Fisher’s exact probability test. *P*-values of less than 0.05 were considered statistically significant.

## Supplementary Information

Supplementary Information 1.
