# Supplementary material for: Streptococcus mutans induces IgA nephropathy-like glomerulonephritis in rats with severe dental caries
Source: Sci Rep. 2021 Mar 11;11:5784. doi: 10.1038/s41598-021-85196-4 (PMC7952735; doi:10.1038/s41598-021-85196-4)
Supplement: Supplementary file 1 — Supplementary Information 1. [file 41598_2021_85196_MOESM1_ESM.docx]

***Streptococcus mutans* induces IgA nephropathy-like glomerulonephritis in rats with severe dental caries**

Shuhei Naka, Kaoruko Wato, Taro Misaki, Seigo Ito, Daiki Matsuoka, Yasuyuki Nagasawa, Ryota Nomura, Michiyo Matsumoto-Nakano, Kazuhiko Nakano

Supplementary Table 1. Serum levels of kidney markers in rats inoculated with Cnm-positive and Cnm-negative *S. mutans*.

| Groups | Serum levels (mean ± SEM) | | |
| --- | --- | --- | --- |
|  | ALB (g/dL) | BUN (mg/dL) | CRE (mg/dL) |
| Control (N=30)  Cnm (-) *S. mutans* (N=18)  Cnm (+) *S. mutans* (N=29) | 3.90 ± 0.04  3.94 ± 0.06  3.90 ± 0.05 | 12.16 ± 0.29  13.12 ± 0.50  12.40 ± 0.38 | 0.38 ± 0.01  0.34 ± 0.01  0.36 ± 0.01 |

Statistical significance was determined using analysis of variance with Bonferroni’s correction. There were no significant differences between the groups. ALB, albumin; BUN, blood urea nitrogen; CRE, creatinine; SEM, standard error of the mean.
